# Extended light exposure increases stem digestibility and biomass production of switchgrass

**DOI:** 10.1371/journal.pone.0188349

**Published:** 2017-11-22

**Authors:** Chunqiao Zhao, Xifeng Fan, Xincun Hou, Yi Zhu, Yuesen Yue, Juying Wu

**Affiliations:** 1 Research & Development Center for Grass and Environment, Beijing Academy of Agricultural and Forestry Sciences, Beijing, P. R. China; 2 Key Laboratory of Urban Agriculture (North), Ministry of Agriculture, Beijing, P. R. China; University of Huddersfield, UNITED KINGDOM

## Abstract

Switchgrass is a photoperiod-sensitive energy grass suitable for growing in the marginal lands of China. We explored the effects of extended photoperiods of low-irradiance light (7 μmol·m^-2^·s^-1^, no effective photosynthesis) on the growth, the biomass dry weight, the biomass allocation, and, especially, the stem digestibility and cell wall characteristics of switchgrass. Two extended photoperiods (i.e., 18 and 24 h) were applied over Alamo. Extended light exposure (18 and 24 h) resulted in delayed heading and higher dry weights of vegetative organs (by 32.87 and 35.94%, respectively) at the expense of reducing the amount of sexual organs (by 40.05 and 50.87%, respectively). Compared to the control group (i.e., natural photoperiod), the yield of hexoses (% dry matter) in the stems after a direct enzymatic hydrolysis (DEH) treatment significantly increased (by 44.02 and 46.10%) for those groups irradiated during 18 and 24 h, respectively. Moreover, the yield of hexoses obtained via enzymatic hydrolysis increased after both basic (1% NaOH) and acid (1% H_2_SO_4_) pretreatments for the groups irradiated during 18 and 24 h. Additionally, low-irradiance light extension (LILE) significantly increased the content of non-structural carbohydrates (NSCs) while notably reducing the lignin content and the syringyl to guaiacyl (S/G) ratio. These structural changes were in part responsible for the observed improved stem digestibility. Remarkably, LILE significantly decreased the cellulose crystallinity index (CrI) of switchgrass by significantly increasing both the arabinose substitution degree in xylan and the content of ammonium oxalate-extractable uronic acids, both favoring cellulose digestibility. Despite this LILE technology is not applied to the cultivation of switchgrass on a large scale yet, we believe that the present work is important in that it reveals important relationships between extended day length irradiations and biomass production and quality. Additionally, this study paves the way for improving biomass production and digestibility via genetic modification of day length sensitive transcription factors or key structural genes in switchgrass leaves.

## Introduction

Switchgrass (*Panicum virgatum* L.) is a perennial erect C_4_ grass naturally ranging from Southern Canada to Central America. Switchgrass exhibits extensive ecoclinal variation across this latitudinal gradient [1]. Switchgrass has been identified as the model crop for US [2], being first introduced in China in the 1980s, where it has demonstrated excellent adaptability to a variety of marginal lands [3]. Switchgrass has shown great potential for producing cellulosic ethanol owing a number of desirable characteristics such as high lignocellulose yield, high adaptability, and improvement of the ecological environment of the marginal lands of China [4].

Flower initiation is driven in many plants via perception of day length changes, a phenomenon known as photoperiodism [5]. Thus, the day length as the light signal is an important factor influencing the vegetative to floral transition of the apical meristem [6], this affecting the duration of the vegetative growth in crops [7]. As is common in traditional C_4_ crops (e.g., *Panicum italicum* L., *Sorghum bicolor* (L.) Moench, and *Zea mays* L.), switchgrass exhibits photoperiod sensitivity [8]. The maximum yield of lignocellulosic biomass (i.e., the main target material) is typically obtained in these cultivars combining extended vegetative growth periods and naturally late-flowering [9, 10]. Consequently, improving the biomass production of switchgrass by delaying its reproductive development via genetic modification of the light signal transduction pathway (day length) seems to be a promising approach.

In the case of traditional C_4_ short-day crops, flowering is usually delayed by extending the photoperiod. Switchgrass ecotypes such as Alamo and Cave-in-Rock (CIR) exhibited delayed development (i.e., delayed panicle emergence or extended panicle duration) by extending the photoperiod to 16 h under a photosynthetic photon flux density of 100 μmol·m^-2^·s^-1^, with a dramatic effect on the biomass production value [11, 12]. According to McMillan et al. [13], no switchgrass clones (originary from Minnesota to Oklahoma, greenhouse at Lincoln, Nebraska) produced normal inflorescences under a 12.5 h light photoperiod. Switchgrass cultivars (origin range 37° N (CIR) to 46° N (Dakota)) showed lower floral development rate and magnitude, with floral initiation and development characteristics comparable to short-day cultivars [14]. However, the studies on photoperiod response in switchgrass have produced inconclusive results. Three upland switchgrass cultivars (i.e., CIR (origin 37°N), Sunburst (origin 42°N), and Dakota (origin 46°N)) were induced to flower under a 24 h low-irradiance (7 μmol·m^-2^·s^-1^) extended daylight photoperiod, and this treatment facilitated seed production [15]. Hence, the effects of light exposure on the growth and development of switchgrass varied largely depending on the different origins, ecotypes, light intensity, and experimental field. The effects of low-irradiance light extended day length periods on the allocation and dry weight of lowland switchgrass Alamo in northern of China remain largely unknown.

Lignocellulosic biomass is an abundant source of carbohydrates from which cellulosic ethanol can be obtained. However, the recalcitrance of the plant cell walls significantly increases biomass processing cost, hindering the conversion and utilization of this resource for the production of ethanol fuel on a large scale [16]. The crystallinity of cellulose, represented by the cystallinity index (CrI), affects negatively to the enzymatic digestibility of biomass, whereas hemicelluloses favor biomass saccharification in *Miscanthus* by decreasing the crystallinity of cellulose [17, 18]. The arabinose (Ara) substitution degree has been revealed as one of the main factors determining the biomass digestibility of energy crops upon various chemical pretreatments [19]. Small wall-networks between monolignols and interlinked-phenolics have been recently reported to affect the biomass enzymatic digestibility in *Miscanthus* [20]. Thus, while coniferyl-lignin (G-lignin) inhibits biomass saccharification to a large extent, the presence of hemicellulosic monosaccharides was found to favor digestibility of Rape seed [21]. Similarly, ammonium oxalate-extractable uronic acids facilitated enzymatic digestibility of *Miscanthus* by reducing the crystallinity of lignocellulose [22]. The different components of the cell walls interact each other generating a complex three-dimensional recalcitrant structure, which affects the digestibility of the cell walls to a different extent. However, little is known about the effect of low-irradiance light extension (LILE) on the cell wall of switchgrass.

This work was aimed to explore the effects of extended day length with low-irradiance light on the biomass dry weight and allocation characteristics of low-land switchgrass Alamo grown in Northern China. In particular, the stem digestibility upon acid or alkali pretreatments and the enzymatic hydrolysis were studied after LILE treatments. Additionally, we studied the characteristics of the stem cell wall components of switchgrass with the aim to gain insight into the effect of LILE on the recalcitrance and digestibility of this cultivar. Although this light extended switchgrass cultivation technology is difficult to scale up, we revealed herein the relationship between extended day lengths and biomass production and quality of switchgrass. Additionally, this study can provide some information to improve biomass production and digestibility via genetic modification (i.e., modifying the day length sensitive transcription factors or key structural genes in the leaves of switchgrass).

## Materials and methods

### Plant material and experimental design

The LILE experiments were carried out on a switchgrass ecotype Alamo planted in a temporarily built plastic greenhouse of the Beijing Academy of Agricultural and Forestry Sciences (39° 34’ N, 116° 28’ E). The light irradiance, temperature, and humidity were uniform in the plastic greenhouse, and no confounding effect originated from the physical position and the light regimen. The average temperature in the greenhouse during the experiment was 22.3°C (highest: 32.4°C, lowest: 15.8°C). The relative humidity in the greenhouse ranged from 50.6 (day) to 89.4% (night). Alamo originary from South Texas (29°N) [23] was introduced to the energy grass basement of the Beijing Academy of Agricultural and Forestry Sciences in 2003. In late May 2013, we planted ca. 200 Alamo seedlings from the seeds to study the biomass production characteristics. Once the switchgrass individuals finished the developmental phases, we cut away the aboveground parts leaving stubbles of ca. 5 cm in height. In late April 2014, we randomly excavated 90 switchgrass plants from the grass basement and each plant was equally separated into three clonal ramets. These ramets were divided into three groups of 90 ramets from the 90 plants. These ramets were transplanted into round plastic basins (25 cm in diameter and 20 cm in height) with three holes at the bottom and placed onto plastic pallets. The cultivation matrix was composed by soil and nutrient soils (1:1, v:v). The final mixed substrate showed a uniform pH of 7.53 and high fertility characteristics (i.e., 55.4 g·kg^-1^ of organic matter, 142 mg·kg^-1^ of available N, 81.3 mg·kg^-1^ of available K, and 22.9 mg·kg^-1^ of available P).

After cultivation for 16 d in the plastic basins (ca. mid-May), we selected 50 ramets from each group to conduct LILE (7 μmol·m^-2^·s^-1^) experiments. The 50 ramets of each group were obtained from the same 50 individuals (i.e., from the same 50 seeds). All the ramets used in this experiment showed a consistent growth status. We subjected one of the groups to a natural light photoperiod (i.e., control experiment). We subjected the remaining two groups either to 18 or 24 h photoperiods, with five replicates for each group (i.e., ten switchgrass plants were considered as one replicate). All these three groups were placed in the same greenhouse. We placed low-irradiance lamps 100 cm above the seedlings to supplement natural light with treatments of 18 or 24 h, according to the sunrise and sunset times. We used a black-out cloth with good air permeability to prevent light disturbance between the different groups. All plants were drip irrigated using tap water as necessary. During the experimental period, we maintained consistent cultivation and management measures for the three groups. The light extension treatments lasted until the sampling time.

### Growth traits and biomass allocation of switchgrass

During the light extension treatments, we surveyed the time at which 80% of the individuals had the panicle emergencies of the first tiller for each replicate and for the three groups. We subsequently averaged the time for the five replicates as the heading start time for each group (i.e., control, 18, and 24 h groups). We harvested the plant samples in early November 2014. The fresh samples were cleaned with tap water and separated into stem, sheath, leaf, panicle, seed, rhizome, and root fractions. All samples were subsequently heated at 105°C for 20 min, dried at 50°C for a long time (ca. 15 d on average) until reaching constant weight (i.e., to avoid any effect of temperature on the cell wall characteristics). After weighing, the stems were smashed with a grinder and the samples from ten individuals were perfectly mixed to form one replicate. The samples (ca. 50 g) were sieved (40-mesh) and stored in plastic automatic sealing bags for digestibility and cell wall studies.

All growth traits were determined the day before sampling. We used a steel tap to determine the height of the plant and the length of the top third leaf (5 replicates), with ten leaves from ten individuals being considered as one replicate. We used an electronic vernier caliper (Mahr Co., Ltd., Suzhou branch, China) to determine the diameter at the middle of the third internodes from the top (5 replicates), with 30 tillers from the ten individuals being considered as one replicate. The basal stems were manually counted to acquire the tiller number (5 replicates), with ten individuals being considered as one replicate.

### Analysis of the digestibility by three methods

We used three treatments (i.e., direct enzymatic hydrolysis (DEH), enzymatic hydrolysis after 1% H_2_SO_4_ (EHAC), and enzymatic hydrolysis after 1% NaOH (EHAL)) to evaluate the digestibility of switchgrass stems. The digestibility experiments were conducted in 15 mL plastic centrifuge tubes following the procedure described by Zhao et al. [24]. We determined the content of hexoses and pentoses following the anthrone/H_2_SO_4_ and the orcinol/HCl methods, respectively, according to the procedure described by Xu et al. [17]. An UV–VIS spectrophotometer (756 PC, Shanghai Hongji Instruments Co., Ltd., Shanghai, China) was used to obtain the absorbance values. Anthrone and orcinol were obtained from Sigma-Aldrich, while ferric chloride was purchased from Sinopharm Chemical Reagent. D-glucose and D-xylose (Sigma-Aldrich) were used as standards to determine the standard curves (R^2^ above 0.9990 were obtained for both hexoses and pentoses). The digestibility experiments were conducted with 5 replicates, with the perfectly mixed sample with ten individuals being considered as one replicate.

### Extraction and determination of non-structural carbohydrates and cell wall components from switchgrass stems

The stem cell wall components were obtained by chemical extraction following the method described by Zhao et al. [24] with minor modifications. After the starch extraction procedure, pectin was obtained using ammonium oxalate (0.5%, w:v). The pectin content was determined by UV–VIS following the procedure described by Huang et al. [25]. Herein, soluble sugars and starches mainly consisted of non-structural carbohydrates. Total lignin was determined by a two-step acid hydrolysis method in accordance with the Laboratory Analytical Procedure of the National Renewable Energy Laboratory [26]. These experiments were conducted with 5 replicates, and the perfectly mixed sample from ten individuals was considered as one replicate.

### Monosaccharide analysis by gas chromatography coupled with mass spectrometry (GC–MS)

Free monosaccharides were released by hydrolysis of the KOH extractable and non-extractable hemicelluloses obtained from the plant cell wall fractions with trifluoroacetic acid (TFA). Monosaccharides were subsequently derivatized to alditol acetates and prepared for GC–MS analysis (Agilent 5977 A GC/MSD) following the procedure described by Xu et al. [17]. Myo-inositol (Sigma-Aldrich) was used as an internal standard. Monosaccharide standards (i.e., L-rhamnose, L-arabinose, L-fructose, D-xylose, D-galactose, D-glucose, and D-mannose) were obtained from Sinopham Chemical Reagent Co., Ltd and used for preparing the calibration curves of all analytes (correlation coefficients over 0.999 in all cases). These experiments were conducted with 5 replicates, and the perfectly mixed sample from ten individuals was considered as one replicate.

### Lignin monomer analysis

The monolignin fraction was determined using the method described by Xu et al., Li et al. and Wu et al. [17, 27, 19]. We used vanillin (G) and syringaldehyde (S, Sinopharm Chemical Reagent) as standard chemicals. Ethyl vanillin was used as an internal standard. The column was placed in a thermostat and operated at 30°C with a CH_3_OH:H_2_O:CH_3_COOH (24:75:1, v/v/v) carrier liquid at a flow rate of 1.1 mL min^-1^. The calibration curves revealed correlation coefficients higher than 0.999 for all the analytes. These experiments were conducted with 5 replicates, and the perfectly mixed sample from ten individuals was considered as one replicate.

### Evaluation of the CrI and cellulose polymerization degree in switchgrass stems

The CrI of the raw materials were determined by X-ray diffraction (XRD, SHIMAZDUOXRD-6100) following the method described by Zhang et al. [18]. An Ubbelohde Viscometer with a 0.88 mm inner capillary diameter (Beijing glass instrument factory) was used to determine the degree of polymerization of the raw cellulose in our plant material following the method described by Li et al. [28]. Copper ethylene diamine (analytical reagent) was provided by the China National Pulp and Paper Research Institute. We calculated the intrinsic viscosity by interpolation using the United States Pharmacopoeia (USP) table (USP35, 2012), which includes intrinsic viscosity and concentration values of the products. These experiments were conducted with 5 replicates, and the perfectly mixed sample from ten individuals was considered as one replicate.

### Data analysis

We conducted significant difference test (one way ANOVA) based on the Duncan method at the *P* < 0.05 and 0.01 levels via SPSS 17. All figures presented in this study were generated with the software Origin 8.5.

## Results and discussion

Switchgrass is native to the North American prairie. Switchgrass is considered as an important biomass energy crop, with the potential to bring great economic and ecological benefits to marginal lands of China avoiding food competition [2, 4]. Switchgrass is sensible to the photoperiod, which mediates the transition from the vegetative to the reproductive development [14, 15]. While LILE is irrelevant to photosynthesis, its effects on biomass allocation (especially biomass digestibility and cell wall) remain largely unknown for switchgrass. In this study, we used low-irradiance light to extend the day length of the switchgrass cultivar Alamo to 18 and 24 h. These experiments were aimed to explore the change in biomass allocation, digestibility quality, and cell wall characteristics as compared to the control switchgrass groups.

### Effects of low-irradiance light photoperiod on the growth and development of switchgrass

The transition from vegetative growth to the sexual growth of short-day plants is significantly affected by the photoperiod [6]. In this sense, photoperiod extension noticeably delayed the flowering time of C_4_ photoperiod sensitive plants such as *Miscanthus sacchariflorus* [29] and energy sorghum hybrids [30]. For switchgrass, CIR, Sunburst, and Dakota (upland ecotypes) exhibited an increased rate and magnitude of floral development under 24 h photoperiod using low irradiance fluorescent bulbs (7μmol·m^-2^·s^-1^) [14]. CIR exhibited the delayed panicle emergency under a 16 h photoperiod with 100 μmol·m^-2^·s^-1^ light compared with the natural photoperiod (12 h day length), while no difference other than the extended panicle duration was found in Alamo [11]. In this study, we found that the 18 and 24 h switchgrass groups headed later (20 and 7 d, respectively) as compared to the natural light photoperiod group, although, unlike previous works, all the switchgrass groups headed finally (Table 1). Thus, extended low-irradiance light day length could act as the light signal and dramatically delay the transition from vegetative to the reproductive growth of low land switchgrass Alamo. These results are consistent with the short day characteristics of low land switchgrass Alamo. The different experimental field might contribute to this result to some degree. However, it is worth noting that the switchgrass Alamo used in this experiment was introduced to Beijing (39°N) from its origin (South Texas 28°), being planted in China for more than 10 years. Thus, the long term acclimation of Alamo can explain the different results obtained.

**Table 1:** Growth characteristics of switchgrass.

Treatments	T1	Plant Height cm	Node number	Tiller number	Leaf Length cm	Stem diameter mm	Sheath length cm	Root length cm	Leaf area cm^2^
Control	55±1.34c	164±1.25b	5.3±0.22b	23.7±0.94B	53.5±1.03a	3.95±0.07b	11.84±0.84B	2828±27b	2502±108B
18 h	75±2.68a	177±2.37a	7.0±0.40a	32.0±1.30A	54.8±1.74a	4.01±0.1b	14.92±0.33A	3188±10a	4014±187A
24 h	62±0.89b	176±1.12a	6.6±0.18a	35.0±1.12A	57.9±1.57a	4.79±0.06a	15.46±0.15A	3014±15a	3572±258A

T1 indicates the time from plantation of switchgrass cultivars to the heading emergency. The switchgrass individuals were light controlled 16 days after plantation. All the data was presented as “means ± SE”, the different small letters followed each column data indicate the significant difference at *P* < 0.05, and the different capital letters followed each column data indicate the significant difference at *P* < 0.01 among the control, 18, and 24 h switchgrass groups.

Extended daylength are reported to delay the reproductive phase, favoring biomass accumulation in *Miscanthus sacchariflorus* [29], Sorghum hybrids [30], and Elephant grass (*Pennisetum purpureum* Schum [31]). Thus, switchgrass cultivar Alamo with extended period of vegetative growth in late flowering types produced a larger amount of leaves [32]. However, the total number of leaves on a tiller was not affected by the extended photoperiod in CIR [11]. Herein, we found that the leaf area of the 18 and 24 h switchgrass groups increased significantly (*P* < 0.01) by 60.43 and 42.77%, respectively (Table 1), for the significantly increased tiller number. Additionally, we found that the 18 and 24 h groups showed significantly increased (*P* < 0.05 or 0.01) plant heights (7.93 and 7.32%), tiller numbers (35.02 and 47.68%), node numbers (32.08 and 24.53%), and root lengths (12.73 and 6.58%), respectively (Table 1). These results indicated that extended low-irradiance light day length during the growth seasons dramatically enhanced the vegetative growth for the low-land switchgrass Alamo.

**Fig 1:**
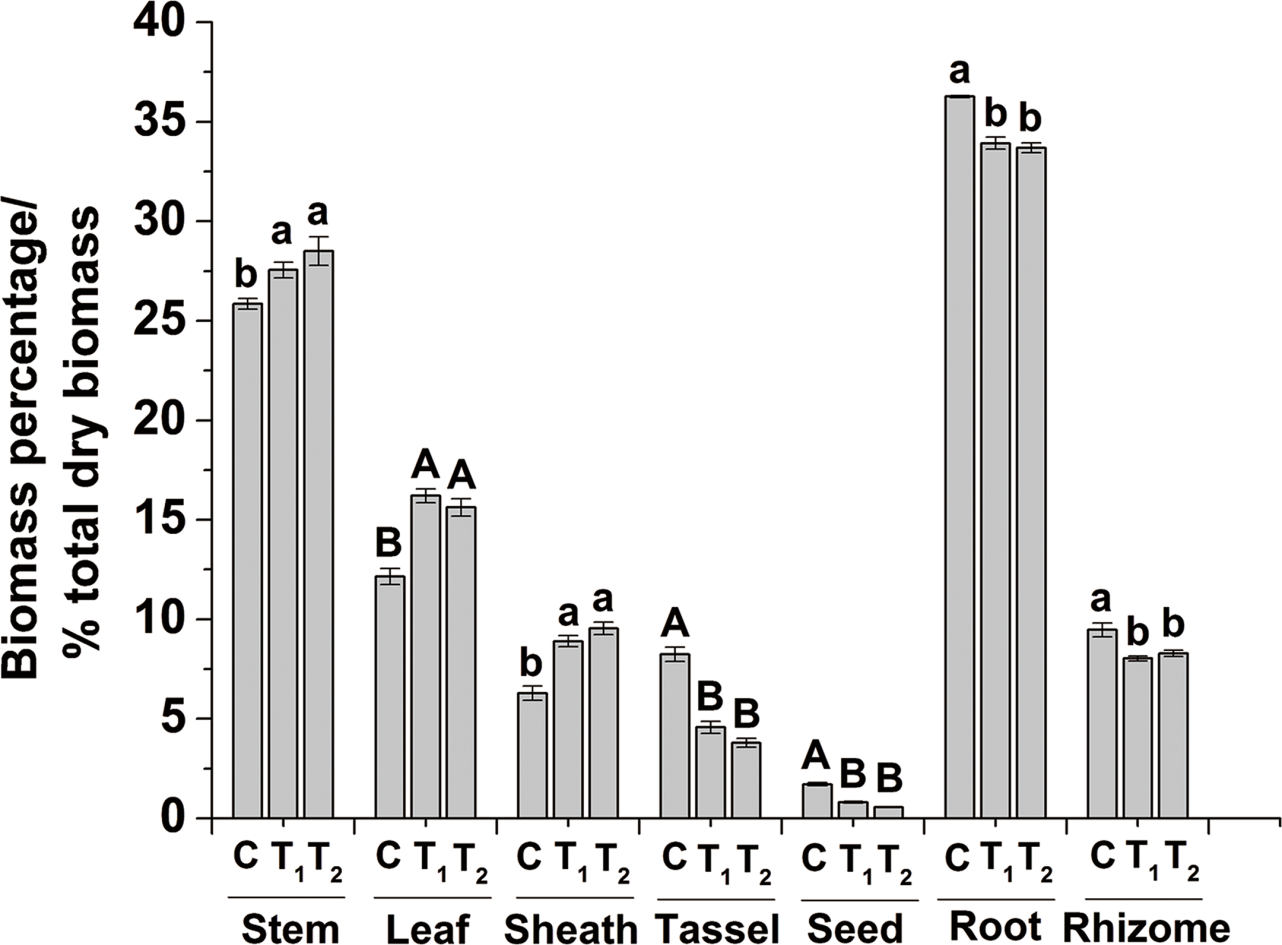
Biomass percentage of different organs in switchgrass with different photoperiods regulated. C indicates the control switchgrass, while T_1_ and T_2_ indicate the switchgrass with extended photoperiods of 18 and 24 h, respectively. The different small letters or capital letters in each group of columns indicate the significant difference at level of *P* < 0.05 or 0.01, respectively. The bar indicates SE (n = 5).

Switchgrass productivity depends to a certain extent on the amount of biomass that can be accumulated before the transition from the juvenile to the adult phases [15]. Herein, we found that LILE (18 and 24 h switchgrass groups) significantly (*P* < 0.01) increased the aboveground biomass dry weight (by 19.97 and 19.46%, Table 2), while noticeably (*P* < 0.01) decreasing those of panicle (by 38.40 and 48.24%) and seed (by 47.97 and 63.51%), respectively (Table 2). In addition, we found that LILE (18 and 24 h) significantly (*P* < 0.05) increased the biomass proportion of stems (by 6.54 and 10.25%, respectively) (Fig 1), thereby mitigating biomass loss owing to the shedding characteristics of leaf and panicle, particularly for late harvest [33]. The significantly (*P* < 0.05) lower stem/leaf ratios of the 18 (by 20.56%) and 24 h (by 14.02%) switchgrass groups (Table 2) might improve the forage quality as a result of the higher contents of crude proteins in leaf. As shown in Fig 1, for both 18 and 24 h switchgrass groups, a significant (*P* < 0.05) decrease was observed in the fraction of dry biomass for the panicle (by 44.42 and 53.88%, respectively) and seed (by 53.49 and 67.44%, respectively), thereby indicating that LILE inhibited sexual reproductive development in Alamo. Thus, LILE could significantly facilitate the accumulation of lignocellulosic materials while controlling the switchgrass invasion on the Chinese marginal lands. Hence, these results provide important information for improving biomass production of switchgrass via genetic modification of the daylength preceptor of leaf, despite this LILE technology is not suitable for large scale applications.

**Table 2:** Biomass dry weight of different switchgrass organs.

Treatments	Stem	Leaf	Sheath	Panicle	Seed	Rhizome	Root	Stem/Leaf	Root cap ratio
		g plant^-1^							
Control	22.24±0.23B	10.45±0.30B	5.43±0.35B	7.11±0.39A	1.48±0.05A	8.13±0.23a	31.21±0.38a	2.14±0.08a	0.84±0.01a
18 h	26.51±0.70A	15.6±0.44A	8.54±0.15A	4.38±0.17B	0.77±0.04B	7.73±0.16a	32.65±0.75a	1.7±0.05b	0.72±0.01b
24 h	27.51±0.64A	15.1±0.54A	9.21±0.28A	3.68±0.06C	0.54±0.03C	8.01±0.20a	32.52±0.08a	1.84±0.09b	0.72±0.01b

All the data was presented as “means ± SE”, the different small letters followed each column data indicate the significant difference at *P* < 0.05, and the different capital letters followed each column data indicate the significant difference at *P* < 0.01 among the control, 18, and 24 h switchgrass groups.

### Effect of low-light photoperiod on the stem components and cell wall characteristics of switchgrass and its impact on digestibility

The plant cell wall is a complicated three-dimensional highly recalcitrant (i.e., hardly digestible) structure. This structure hinders the accessibility to the sugars embedded in plant cell walls, complicates biomass processing, and increases costs [34, 35]. The digestibility of lignocellulosic materials affects significantly the conversion into ethanol. This parameter has been typically measured by calculating the sugar yield (% dry matter or cellulose) released after mildly acidic or alkaline pretreatments followed by enzymatic hydrolysis [17, 18, 20, 24]. Light intensity has been reported to positively affect *in vivo* digestibility by sheep in ryegrass (*Loliurnperenne* L.) [36, 37]. However, little is known about the effects of LILE on the digestibility and cell wall characteristics of switchgrass stems. In this study, we used three methods (i.e., DEH, EHAC, and EHAL) to analyze the digestibility of switchgrass stems. We found that the sugar yield efficiencies after DEH, EHAC, and EHAL treatments significantly (*P* < 0.05 or 0.01) increased versus the control group (Table 3). Hence, the LILE treatment may have caused some changes in the composition or cell wall characteristics of switchgrass stems.

**Table 3:** Stem digestibility of switchgrass using 3 pretreatment methods.

Digestibility procedure		Types	Sugar content/ % dry matter		
			Control	18 h	24 h
Direct enzymatic hydrolysis		C5	3.65±0.12a	3.84±0.18a	3.39±0.13a
		C6	8.2±0.37B	11.81±0.47A	11.98±0.40A
		Total	11.85±0.49B	15.65±0.53A	15.37±0.40A
1% H_2_SO_4_	Pretreatment	C5	16.15±0.76a	15.98±0.12a	15.3±0.36a
		C6	6.07±0.16b	7.94±0.13a	7.11±0.25a
	Enzymatic hydrolysis	C5	3.08±0.13a	3.23±0.13a	3.14±0.09a
		C6	8.41±0.25B	11.44±0.31A	12.03±0.33A
	Total	C5	19.23±0.89a	19.21±0.25a	18.44±0.45a
		C6	14.48±0.41B	19.38±0.43A	19.14±0.59A
1%NaOH	Pretreatment	C5	3.17±0.09a	2.91±0.21a	2.98±0.17a
		C6	3.03±0.13c	3.97±0.12a	3.64±0.11b
	Enzymatic hydrolysis	C5	24.41±0.53C	31.2±0.40A	27.78±0.43B
		C6	20.66±1.13B	26.95±0.39A	27.59±0.38A
	Total	C5	27.57±0.62c	34.11±0.60a	30.75±0.60b
		C6	23.69±1.25B	30.93±0.51A	31.23±0.49A

All the data was presented as “means ± SE”, the different small letters followed each row of data indicate the significant difference at *P* < 0.05, and the different capital letters followed each row of data indicate the significant difference at *P* < 0.01 among the control, 18, and 24 h switchgrass groups.

Non-structural carbohydrates (NSCs) in crop stems are closely related to yield stability and stress tolerance. NSCs have a strong influence on the biofuel production of energy crops [38]. In the case of the LILE treated switchgrass groups studied herein, the NSCs (i.e., starch and soluble sugar) were found to be preferentially stored in storage organs such as stems, roots, and rhizomes (Table 4). Notably, the starch and soluble sugar contents of stems were significantly (*P* < 0.05) higher than those of the control switchgrass group (Table 4). This might be caused by a delayed transition from juvenile to adult phase in switchgrass, as shown in the genetically modified [39] and panicle removed [24] switchgrasses. This could be the main factor increasing the hexoses yields of the stem after NaOH and H_2_SO_4_ pretreatments (Table 3), since only minor amounts of hexoses were extracted from cellulose after mild acidic or alkaline pretreatments.

**Table 4:** Content of the NSCs in different switchgrass organs.

Treatments	Leaf		Stem		Root		Rhizome	
	Soluble sugar	Starch	Soluble sugar	Starch	Soluble sugar	Starch	Soluble sugar	Starch
Control	1.55±0.10a	2.35±0.15B	2.06±0.06B	3.69±0.20B	2.25±0.09b	8.63±0.30C	2.64±0.25B	9.11±0.40B
18 h	1.68±0.07a	3.49±0.17A	3.15±0.13A	4.77±0.33A	3.19±0.16a	10.36±0.37B	3.57±0.32A	13.66±0.47A
24 h	1.59±0.11a	3.94±0.18A	3.29±0.13A	4.96±0.37A	3.55±0.18a	13.67±0.41A	3.67±0.14A	14.56±0.69A

The different small letters followed each column data indicate the significant difference at *P* < 0.05, and the different capital letters followed each column data indicate the significant difference at *P* < 0.01 among the control, 18, and 24 h switchgrass groups, all the data were presented as “means ± SE”.

**Fig 2:**
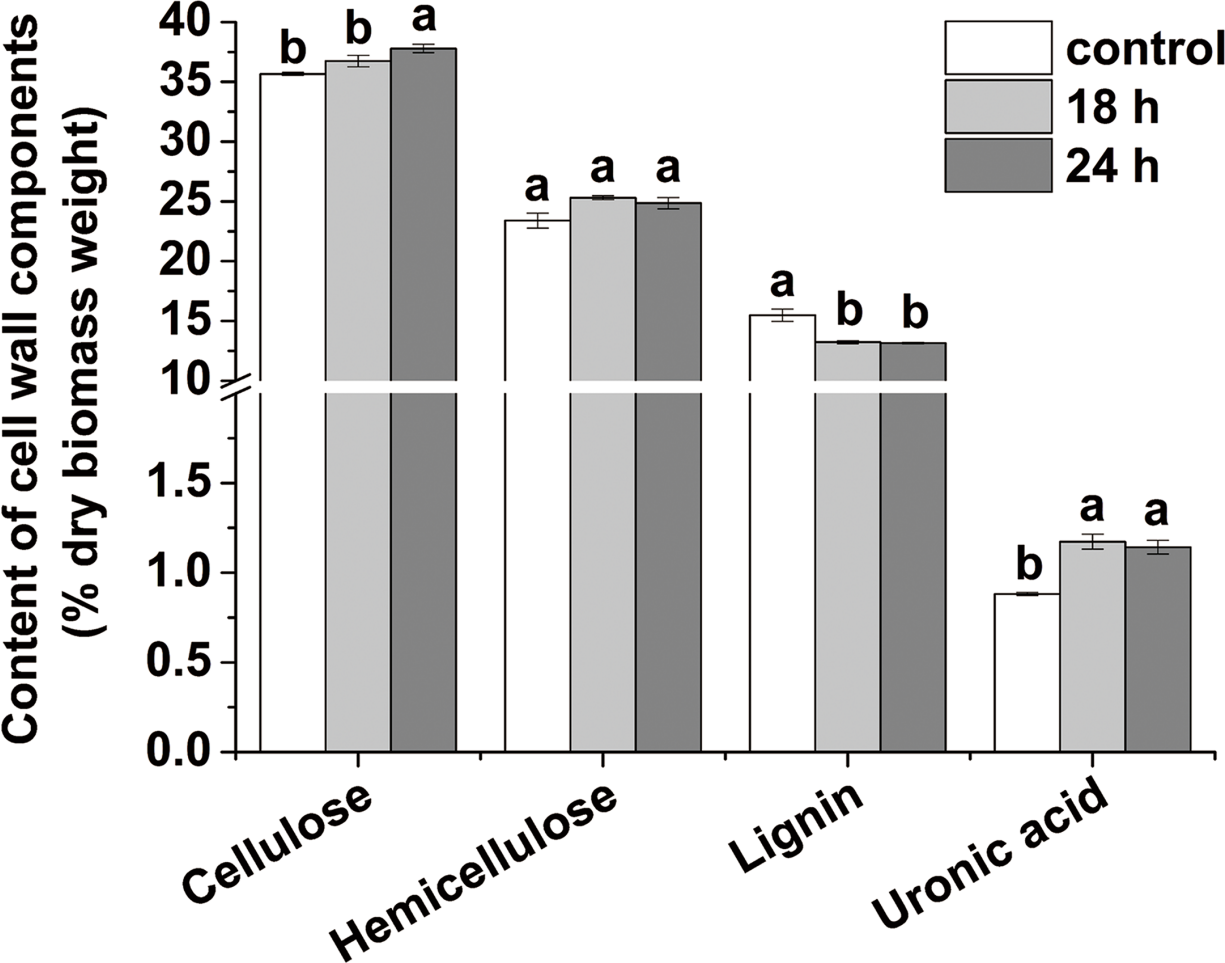
Content of cell wall components in switchgrass stem. The different small letters or capital letters in each group of columns indicate the significant difference at level of *P* < 0.05 or 0.01, respectively. The bar indicates SE (n = 5).

**Fig 3:**
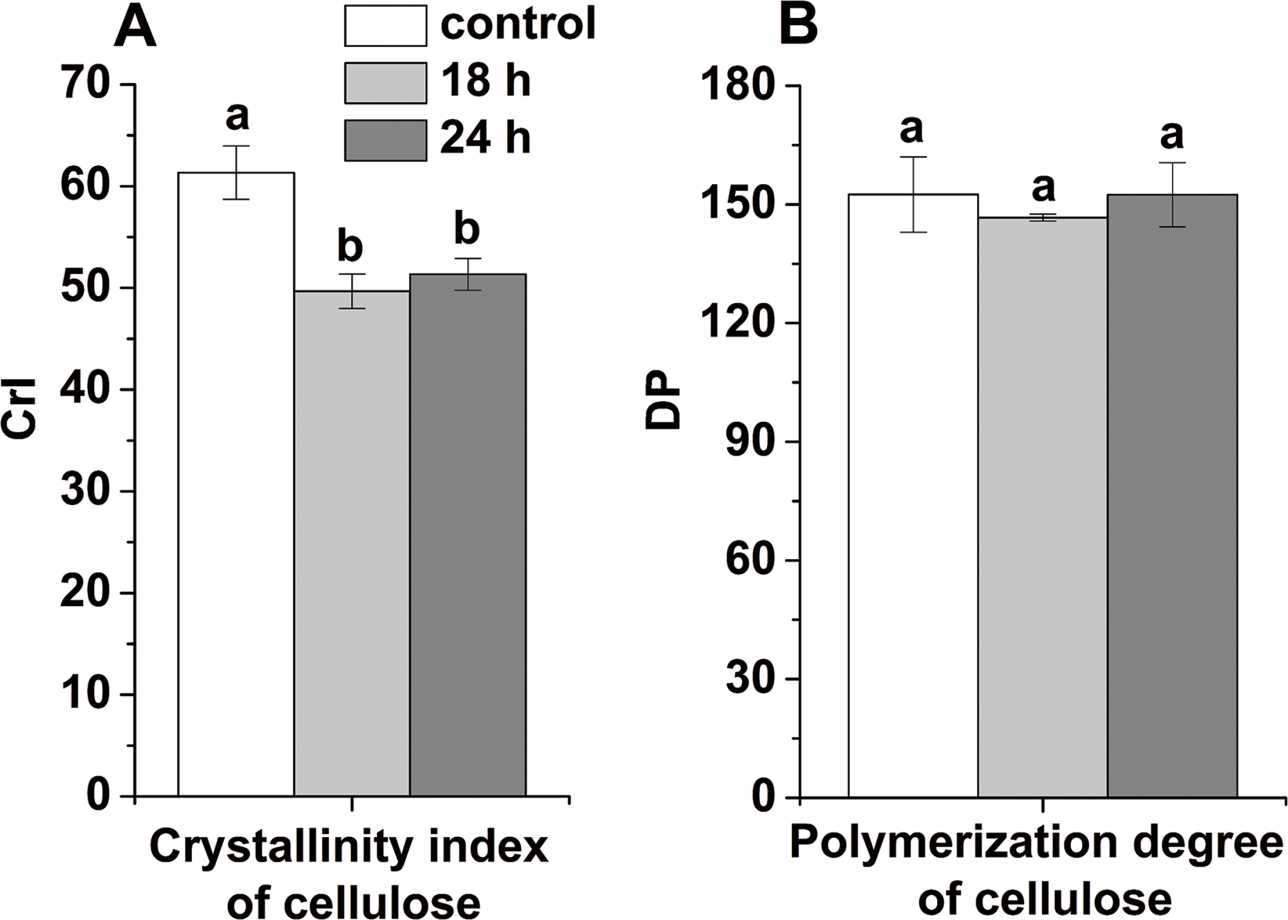
Cellulose characteristics in the switchgrass stem. (a) Cellulose crystallinity indexes, and (b) depolymerization degree of cellulose in the switchgrass stem. The different small letters in each group of columns indicate the significant difference at level of *P* < 0.05 or 0.01, respectively. The bar indicates SE (n = 5).

**Fig 4:**
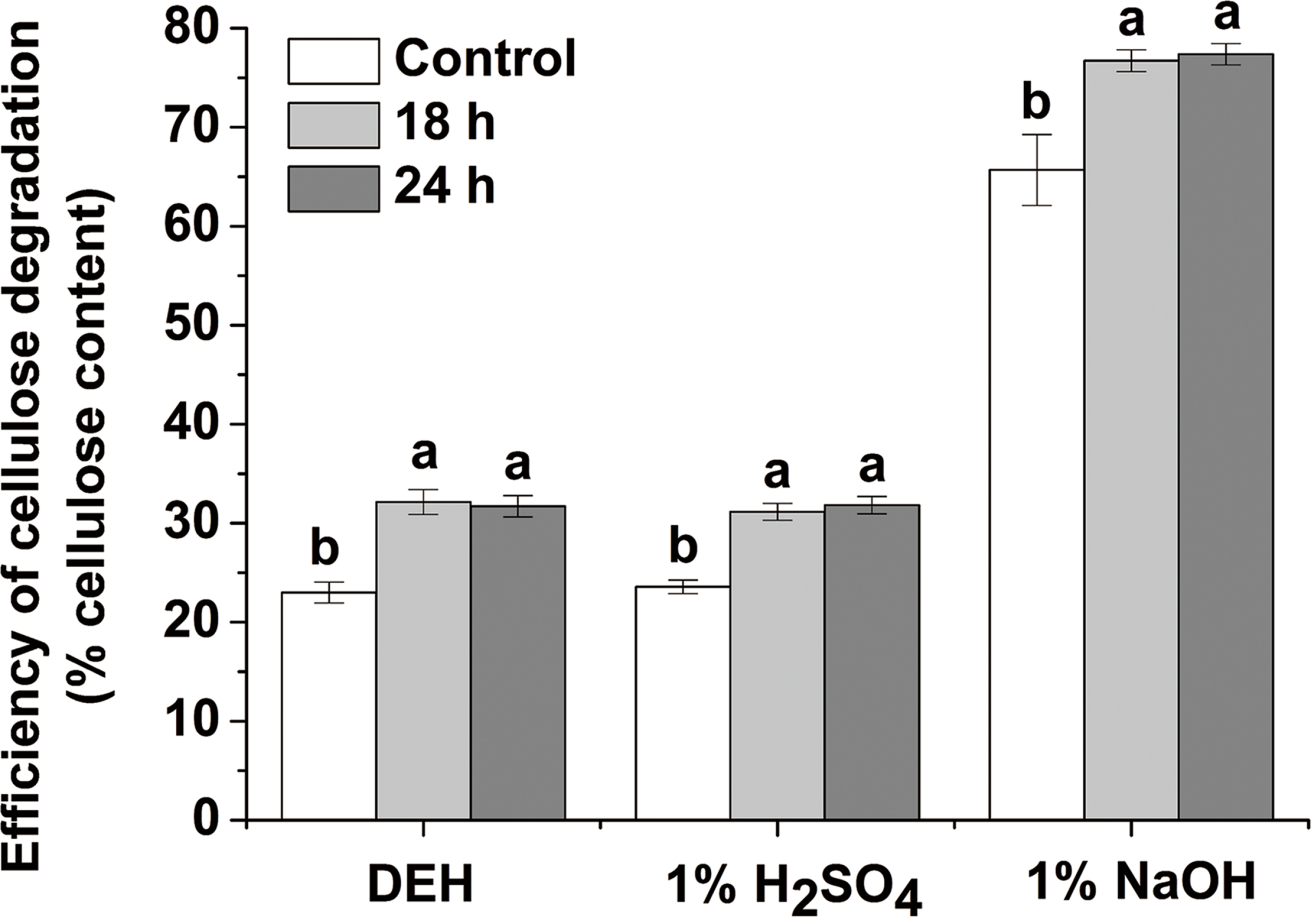
Cellulose degradation efficiency of switchgrass stem. The efficiency was calculated as the fraction of hexoses from the enzymatic hydrolysis procedure to the total cellulose in this study. The different small letters or capital letters in each group of columns indicate the significant difference at level of *P* < 0.05 or 0.01, respectively. The bar indicates SE (n = 5).

Cellulose is one of the most abundant carbohydrates on Earth. Large amounts of liquid fuels could be derived from this polymer. Phillips et al. [40] reported that long day lengths could increase the cellulose contents of forage grasses. Bowman and Law [41] found that the day length affected negatively to the fraction of cellulose in *Dactylis glomerata* L. and *Bromus inermis* Leyss subjected to an 18 h photoperiod versus a shorter period (14 h). While photoperiod has minor effects on chemical composition of timothy (*Phleumpratense* L.) [42]. In this study, we found that the cellulose content of stems significantly (*P* < 0.05) increased (by 5.97%) only in the case of the 24 h switchgrass groups (Fig 2). A higher content of the desired cellulose in lignocellulosic biomass is expected, increasing the amount of sugars that can be obtained. The difference between 18 h and 24 h switchgrass groups in the cellulose content still needs further study. Cellulose is a high molecular weight linear polymer composed of D-glucopyranose units linked by β-1, 4-glycosidic bonds. It typically contains hydrophobic (i.e., crystalline) and hydrophilic (i.e., amorphous) regions [43]. The CrI and the degree of polymerization (DP) are the main factors hindering the enzymatic hydrolysis of cellulose [44]. CrI of both photoperiod-regulated switchgrass groups (18 and 24 h) significantly (*P* < 0.05) decreased by 19.01 and 16.31%, respectively (Fig 3). These results might be the key factor explaining the higher hexoses yield (Table 3) and cellulose degradation efficiencies (Fig 4) obtained after the three treatments. At the transcriptional level, different light signals influenced the expression of CesA genes and members of the cellulose synthase-like families in both Arabidopsis and Zea mays [45]. Differential expression of cellulose synthase genes might result in the random arrangement of glucan chains rendering more amorphous regions in the cellulose chains. This facilitated the accession of the cellulase to the glucan chains improving the hexoses yields and cellulose degradation efficiency of switchgrass stems.

Hemicellulose is a polysaccharide of plant cell walls comprised of various sugar units with β-(1→4)-linked backbones with an equatorial configuration [46]. The stems of LILE-treated switchgrass groups showed similar hemicelluloses contents than the control group (Fig 2), in line with the minor (*P* > 0.05) differences in the pentose yields of stems after EHAC and EHAL versus control (Table 3). This result indicated that the LILE treatment only slightly affected the hemicelluloses content of low land switchgrass Alamo. In the case of secondary cell walls of Gramineae plants, the major hemicellulose component (i.e., xylan) is most commonly substituted by α-L-arabinofuranosyl units at the C2- or the C3- positions in arabinoxylan, and by α-D-glucopyranosyluronic units or their 4-O-methylderivative side chains at the C2-position in glucuronoarabinoxylan (GAX) [47]. Recently, arabinose (Ara) substitution in xylan, an indicative of the branched structure of hemicellulose, was reported to positively affect biomass digestibility by decreasing the cellulose CrI in *Miscanthus* [17]. In this work, we found that the contents of rhamnose (Rha), Ara, and galactose (Gal) in stems significantly (*P* < 0.05 or 0.01) increased for both photoperiod-regulated switchgrass groups (Table 5). The mannose (Man) content in hemicelluloses of stems significantly (*P* < 0.05 or 0.01) increased for the 18 and 24 h switchgrass groups by 54.55 and 95.45%, respectively (Table 4), while the Xylose (Xyl) content decreased significantly (*P* < 0.05) by 5.95 and 7.03%, respectively (Table 5). Thus, the Xyl/Ara ratio of hemicelluloses for both photoperiod-regulated switchgrass group stems significantly (*P* < 0.05) decreased by 20.78 and 24.03%, respectively (Table 5). Uronic acids of AO-extracted components favored biomass digestibility by reducing the cellulose crystallinity in *Miscanthus* [22]. The content of AO-extracted uronic acids of stem significantly (*P* < 0.05 or 0.01) increased as compared to the control group for both photoperiod-regulated switchgrass groups (Fig 2). Hence, we concluded that LILE (18 and 24 h) dramatically increased the degree of Ara substitution and AO-uronic content, both dramatically decreasing the CrI of cellulose forming part of the stems. As a result, digestibility was enhanced facilitating the subsequent conversion and utilization of switchgrass biomass for the production of hexoses. The presence of Man, Gal, and Rhamoieties in the hemicelluloses might also play a positive role in the observed improved stem digestibility of switchgrass.

**Table 5:** Monosaccharide composition of hemicellulose in switchgrass stem.

Monosaccharides Content (%)	Control	18 h	24 h
Rha	0.22±0.01B	0.32±0.01A	0.3±0.01A
Ara	10.12±0.41B	14.17±0.54A	14.96±0.76A
Xyl	87.52±1.35a	82.31±1.08b	81.37±1.40b
Man	0.22±0.01C	0.34±0.01B	0.43±0.01A
Glu	1.36±0.03a	1.43±0.02a	1.43±0.02a
Gal	1.22±0.04B	1.43±0.05A	1.51±0.06A
Xyl/Ara	7.99±0.24A	6.33±0.35B	6.07±0.41B

Rha: rhamnose, Ara: arabinose, Xyl: xylose, Man: mannose, Glu: glucose, Gal: galactose, all the data was presented by mean of “means ± SE”, the different small letters followed each row of data indicates the significant difference at *P* < 0.05, and the different capital letters followed each row of data indicates the significant difference at *P* < 0.01 among the control, 18 h and 24 h switchgrass groups.

Lignin is considered as the main barrier avoiding cellulose digestibility [48]. The percent lignin of *Dactylis glomerutu* L. and *Bromtls inermis* Leyss' showed no correlation with the day length [41]. However, we found that the lignin contents of stems significantly (*P* < 0.05) decreased for both (18 and 24 h) groups (Fig 2), which largely contributed to the increased sugar yields of the stems (Table 3). Lignification of plant cell walls increased drastically from the vegetative to the reproductive stages [49]. The control of the transition from vegetative to reproductive phases improved the biomass yield and simultaneously reduced the lignin content in *Medicago truncatula* [50]. Hence, the main reason for the decreased lignin content in LILE treated switchgrass stems was the delayed transition from vegetative to reproductive phases, caused by the extended day length. Three monomer units (i.e., H, G, and S) constitute the complex racemic heteropolymer structure of lignin and the relative ratios of these components (especially the S/G value) have been reported to dramatically affect the digestibility of the lignocellulosic material [20, 28, 51]. Herein, the S- and G-lignin contents (% dry matter) for both LILE treated switchgrass stems significantly decreased as compared to the control group, in agreement with the trend of the total lignin content. The S/G ratio significantly (*P* < 0.05) decreased by 15.38 and 16.67% for the 18 and 24 h switchgrass groups, respectively (Table 6). These lower S/G ratios could positively affect the stem digestibility in switchgrass, in line with previous results [24]. It has been reported that the relative S lignin content and the S/G ratio increases as the plants mature [49]. G-lignin seems to be deposited at the early stages of plant growth, while S lignin was preferentially formed at the later developmental stages [49]. As described above, both photoperiod-regulated switchgrass groups exhibited delayed reproduction development versus the control group. Thus, LILE dramatically affected the deposition of different lignin monomers in switchgrass stems by significantly delaying the transition from the vegetative to the reproductive development states, this finally leading to stems with higher sugar yields.

**Table 6:** Content of lignin monomers (% dry matter) in switchgrass stem.

Treatments	Lignin monomers		S/G
	G	S	
control	262±7A	203±4A	0.78±0.02A
18 h	219±5B	145±3B	0.66±0.01B
24 h	217±4B	141±2B	0.65±0.02B

G: coniferyl alcohol, S: sinapyl alcohol, the different capital letters followed each column data indicates the significant difference at *P* < 0.01 among the control, 18 h and 24 h switchgrass groups, all the data was presented by mean of “means ± SE”.

Light exposure is a very important signal triggering or delaying sexual development in plants such as heading and flowering. In this study and our prior work [24], we found that panicle removal or LILE dramatically delayed or even inhibited the reproductive development of switchgrass, distinctively improving biomass production and digestibility by increasing NSCs content and altering fine structure of hemicelluloses and lignin. As a result, the CrI in switchgrass was reduced. The cell wall components and structure (e.g. lignin deposition) were closely related to the development phase of the plant. Delaying the transition from vegetative to reproductive development via genetic modification in the day length signal perceptor might be a promising strategy simultaneously improving biomass production and quality.

## Conclusions

The following conclusions were obtained from this study:

1. Extending daylength with low-irradiance light delayed heading and enhanced vegetative growth of low-land switchgrass ecotype Alamo, increasing the dry weight of the vegetative organs while decreasing the weight of sexual organs.
2. We found improved stem digestibility for the 18 and 24 h switchgrass groups after DE, EHAC, and EHAL treatments.
3. The LILE treatment significantly increased the NSCs content while notably reducing both the lignin content and the S/G ratio, significantly enhancing the stem digestibility as a result. Remarkably, LILE significantly decreased the CrI of cellulose by increasing the Ara substitution degree in xylan as well as the content of ammonium oxalate-extractable uronic acids, both facilitating cellulose digestibility.
